# Unilateral proptosis in thyroid eye disease with subsequent contralateral involvement: retrospective follow-up study

**DOI:** 10.1186/1471-2415-13-21

**Published:** 2013-05-30

**Authors:** Diego Strianese, Raffaele Piscopo, Andrea Elefante, Manuela Napoli, Chiara Comune, Immacolata Baronissi, Raffaele Liuzzi, Mariantonia Ferrara, Alessia D’alessandro, Pasquale Ruggiero, Pasquale Napolitano, Piergiacomo Grassi, Adriana Iuliano, Carmela Russo, Arturo Brunetti, Giulio Bonavolontà

**Affiliations:** 1 Departement of Visual Science, University of Naples “Federico II”, Naples, Italy; 2 Department of Diagnostic Imaging, University of Naples “Federico II”, Naples, Italy; 3 Institute of Biostructure and Bioimaging, National Research Council of Italy (CNR), Naples, Italy

**Keywords:** Thyroid eye disease, Unilateral proptosis, Exophthalmos

## Abstract

**Background:**

The purpose of this retrospective follow-up study is to evaluate the prevalence of patients with thyroid eye disease presenting with apparent unilateral proptosis and determine the occurrence of exophthalmos in contralateral non-proptotic eye over the time. Associated features with this event were evaluated.

**Methods:**

A cohort of 655 consecutive patients affected by thyroid eye disease with a minimum follow-up of 10 years was reviewed. Exophthalmos was assessed by using both Hertel exophthalmometer and computed tomography (CT). The influence of age, gender, hormonal status and of different therapies such as corticosteroids, radiotherapy and surgical decompression on this disease progression was evaluated.

**Results:**

A total of 89 patients (13.5%) (95% confidence interval [CI] 15%-10%) had clinical evidence of unilateral exophthalmos at the first visit. Among these, 13 patients (14%) (95% CI 22%-7%) developed subsequent contralateral exophthalmos. The increase of protrusion ranged from 2 to 7 mm (mean of 4.2). The time of onset varied from 6 months to 7 years (mean time: 29 months). Smoking status, young age and surgical decompression are significantly associated with development of contralateral proptosis (p< .05).

**Conclusions:**

Asymmetric thyroid eye disease with the appearance of unilateral exophthalmos at the initial examination is a fairly frequent event, while subsequent contralateral proptosis occurs less commonly. However, physicians should be aware that young patients, particularly if smokers, undergoing orbital decompression in one eye may need further surgery on contralateral side over time.

## Background

Thyroid Eye Disease (TED) is the most frequent extrathyroidal manifestation of Graves’ disease [1], and results from an increased volume of orbital tissues (connective and adipose tissue, interstitial enlargement of extraocular muscles) within the enclosed space of the bony orbits [2]. Although excellent review articles have been published on TED [1,3-5], it remains a pathogenetic enigma and a therapeutic dilemma. Once initiated, the orbital immune process frequently assumes a momentum of its own, leading to non-specific but nonetheless harmful consequences such as tissue hypoxia, oxygen free radical damage, and fibrogenic tissue remodeling [6].

Thyroid Eye Disease is a self-limiting disease with active and static phases. The initial active inflammatory phase usually lasts for 6–24 months, but may sometimes continue for several years [2,7,8].

Exophthalmos, probably the most widely known symptom of TED, occurs in 20–30% of patients with Graves’ disease and up to 40–70% of patients with TED [9]. In the majority of patients both eyes are equally affected, although often in an asymmetric manner [10]. Pure unilateral ophthalmopathy is rare, with 5% to 11% of cases showing no progression to bilateral disease [11]. However, the literature documenting unilateral TED is weak and probably patients presenting with very asymmetric TED show subclinical disease in the less affected orbit. Radiological findings can often be demonstrated in the fellow eye and clinical evidence of TED may become clear during the course of the disease [8]. The natural history of unilateral TED is unknown and the diagnosis remains challenging [12]. This study aims to assess the patients who were referred to a tertiary orbital clinic with TED with the appearance of unilateral proptosis, and how many in this group went on to develop subsequent contralateral involvement. The influence of age, gender, hormonal status and of different therapies such as corticosteroids, radiotherapy and surgical decompression on this phenomenon is evaluated.

Given that TED is a disfiguring and disabling disease that influences and impairs the quality of life of affected individuals [3], when contralateral proptosis occurs, the need for further therapy is a frustrating event for the patient. To our knowledge, there are no specific reports in the literature on this phenomenon, and its incidence and characteristics are poorly documented.

## Methods

A cohort of 655 consecutive patients with TED who visited and were managed at the Orbital Unit of the Department of Visual Science of the University of Naples “Federico II” from January 1995 to December 2009, were retrospectively reviewed. The aim was to determine the percentage of patients presenting with unilateral TED with the appearance of unilateral exophthalmos and who had previous clinical history with no remarkable changes in their appearance and to evaluate the percentage of patients who developed over time exophthalmos on contralateral non-proptotic eye. To ensure a reasonable time period that produces suitable data for statistical analysis, the minimum follow-up time for the patients was 10 years. Data was extracted from a special TED form which is routinely completed for each patient in our Orbital Unit. The study adheres to the declaration of Helsinki. The form included different sections regarding age, sex, thyroid gland disorder, associated systemic diseases, medication history, and eye symptoms at presentation of TED. It also included a section for visual function tests including best corrected visual acuity, optic disc, color vision, visual field, intraocular pressure (IOP) in primary and up-gaze, Hertel exophthalmometry, eyelid examination, ocular motility examination [13], slit-lamp examination and findings on orbital imaging. The examination included a clinical activity score (CAS) and a NOSPECS severity score [14]. In the case of asymmetric severity and/or activity scores, the worst score was considered for statistical analysis. To help enhance the accuracy of data collection, the charts of patients with more complicated medical courses were independently abstracted by senior a ophthalmologist who is experienced in the care of patients with TED (DS, RP, GB), and by an ophtlhalmologist-in-training (CC, PG, IB).

Clinical measurement of the exophthalmos is performed in our clinic by using "Hertel" Oculus exophthalmometer, and followed by using a similar intercanthal width for each individual patient. Two observers routinely perform readings: usually one fellow or resident and one senior (DS, RP, GB). An asymmetric proptosis was considered significant if the difference between eyes was > 2 mm [7], and when measurement of the non-proptotic eye produced a result of < 16 mm for female and <20 mm for males according to the previous study on Caucasian population performed by Mourits [15].

For the study purposes the exophthalmos was quantified by reviewing the CT scan. All patients referred to our clinic presenting with exophthalmos routinely undergo CT evaluation to exclude the presence of other causes of proptosis and to assess the follow-up [16,17]. In this cohort, 353 patients (54%) were evaluated with a CT scan (Marconi MX 8000, Philips Medical System) performed at the Radiology Department of the University of Naples “Federico II”. Standard axial, 1.33-mm-thick, spiral CT images (increment 0.6mm, pitch 0.875 mm, mAS/slice 200, KV 120) were obtained. Following acquisition, the scans were post-processed with multiplanar reconstructions (MPR) on the coronal plane. The window settings were adjusted to better visualize the single structures (muscles, globe, nerves, and fat). Upon admission, 302 patients (46%) produced a CT exam which had been previously conducted at another medical center; the exams were deemed appropriate for this study if performed with 1.33-mm-thick acquisition. The authors (AE, MN) had post-processed the DICOM files images with multiplanar reconstructions (MPR) on the coronal and sagittal plane.

Results of the examinations were scored by the same observer (AE, MN) in a blinded manner and initial and long-term follow-up CT examinations were digitally combined and compared to determine variations of the following parameters: side of involvement (L/R); number of involved EOMs; most severely affected EOM; degree of exophthalmos; apical crowding (Y/N); optic nerve (ON) rectitude (Y/N); superior ophthalmic vein (SOV) enlargement (Y/N); orbital lipomatosis and adipose involution of EOMs (Y/N). The lengths of the interzygomatic line and globe position were measured on axial scans of the mid-globe section. The number and dimension of the involved EOMs were evaluated on CT coronal images [18,19]. Volumetric analysis was eventually performed. To determine the position of the globe and the grade of the exophthalmos, on CT, the perpendicular distance between the interzygomatic line and the posterior margin of the globe at the mid-globe section (where the middle portion of the nerve was visualized), was measured on an axial scan.

Medical and/or surgical treatments administered for the initial proptosis were carefully analyzed to study their influence on the outcome. Thyroid function- status was based on standard test.

To evaluate the influence of age, gender, hormonal and smoker status and of different therapies such as corticosteroids, radiotherapy and surgical decompression on this phenomenon on the development of contralateral proptosis, we compared statistically the group of patients who remained with asymmetric proptosis (group 1) with those who showed contralateral proptosis over time (group 2).

Univariate analysis was used to evaluate correlations between clinical factors (sex, age, surgery, corticosteroid therapy, radiotherapy, thyroid hormonal status) and the incidence of controlateral disease. Dichotomic variables were tested by Pearson Chi-square test, Fischer exact test or Students’T test when appropriate. The median and the range were used to describe all continuous variables and nonparametric techniques were used for analyzing them (Mann–Whitney U test). To evaluate the risk associated with the presence of a significant clinical factors the Cox regression analysis was adopted. All statistical tests were two-sided, and a p value of 0.05 was considered statistically significant. Statistical analysis was performed with SPSS 18.0 statistical software (SPSS Inc., Chicago, IL).

## Results

Of the 655 patients enrolled in this study, 89 (13.5%) (95% confidence interval [CI] 15%-10%) presented with unilateral exophthalmos. Exophthalmometer values in the proptotic eye ranged from 17 to 29 mm, with a median value of 25.2. There were 31 (35%) men and 58 (65%) women. CAS values ranged from 3 to 6 with a mean value of 4.1 in the proptotic eye and from 2 to 4 with a mean of 2.8 in the fellow eye. No significant relation was found between the CAS at the initial visit and the development of proptosis (worst score was considered for the analysis in asymmetric cases). Twenty-four (27%) patients had concomitant eye movement restriction in the proptotic eye; none had extraocular muscle impairment in the unaffected eye. Among the 89 patients with asymmetric proptosis, 13 patients (14%) (95% CI 22%-7%) (7 women and 6 males) developed contralateral exophthalmos during the 10-years of follow-up. In all cases, the increase in Hertel exophthalmometry measurements was > 2 mm, and ranged from 2 to 5 mm (median of 3.2). Review of the CT scan confirmed this data and differences were not statistically significant. The contralateral exophthalmos developed over a length of time, which varied from a minimum of 6 months to a maximum of 7 years, with a median time of 29 months. In particular, contralateral proptosis developed in 3 (23.%) patients within 36 months from the onset (3±0.9-mean CAS ± standard deviation), in 4 patients (30%) between 3 to 5 years (4.41±1.3 mean CAS ± standard deviation), and in 5 patients (37%) at a time >5 years (5±0.786 mean CAS ± standard deviation).

**Figure 1:**
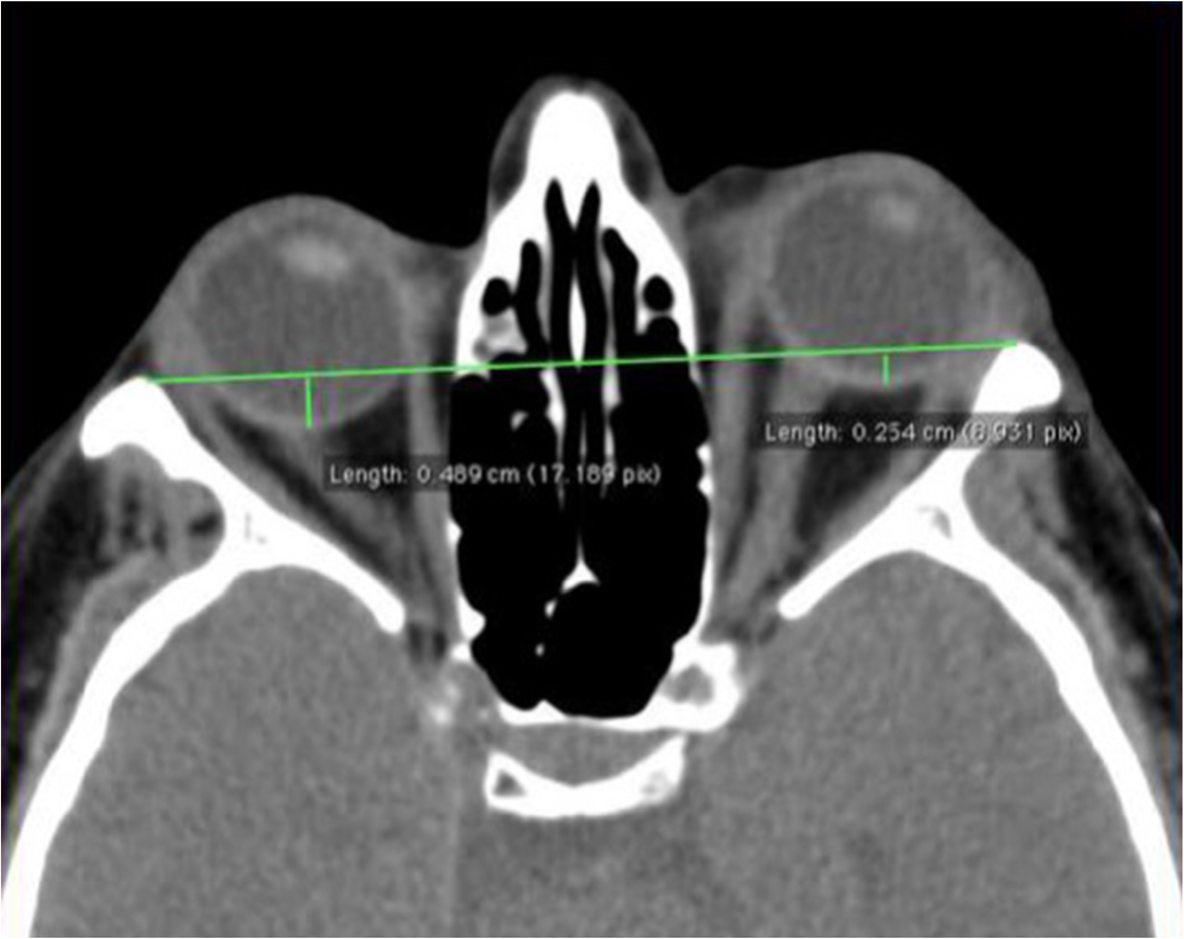
**Axial 1.3 mm CT scan of the orbit in soft-tissue windows.** Unilateral exophthalmos is evaluated by the perpendicular distance between the interzygomatic line and the posterior margin of the globe at the mid-globe sections.

Volumetric analysis of proptotic eye on CT performed at the time of initial presentation showed in proptotic eyes: enlargement of both the orbital fat compartment and the extraocular muscles in 56 patients (63%), involvement of only adipose tissue in 14 patients (16%) and increased enlargement of only extraocular muscle (mean sizes: inferior rectus 6.5 mm, superior rectus 5.8 mm, medial rectus 4.6 mm, and lateral rectus 5.1 mm) in 19 patients (21%) whereas in non-proptotic eyes: absent in 56 patients (63%) or minimal increase (37%) of the fat compartment with muscles of normal size (79%) or minimum enlargement of one muscle (21%) (mean size: inferior rectus 4.3 mm, superior rectus 5.0 mm, medial rectus 4.0 mm, and lateral rectus 3.6 mm); these features were consistent with the condition of asymmetric proptosis (Figure 1).

Imaging at the time of development of contralateral exophthalmos revealed increases in both the individual and total sizes of the extraocular muscles (mean size: inferior rectus 6.3 mm, superior rectus 5.9 mm, medial rectus 4.8 mm, and lateral rectus 5.0 mm) using the previously described criteria [20]. Distribution of the patients in the 2 groups was as follows: group 1, 76 patients and group 2, 13 patients. Patients who developed contralateral exophthalmos (group 2) were an average of 8.5 years younger than those patients who remained stable (group 1) (p = 0.039).

More patients who showed unilateral proptosis were smokers compared to those who were not (chi-square test, *p* = 0.021).

Different therapies were administrated to the 2 groups. Group 1: 40 patients (52.%) received corticosteroid pulse therapy, 4 patients (5.%) received radiotherapy, and 22 patients (28.%) received orbital decompression. Group 2: 10 patients (76.%) received corticosteroid therapy, 8 patients (61.%) received orbital decompression, and 1 patient (7.%) received radiotherapy (Table 1). Among those different therapies, only surgical decompression was related with a higher incidence of contralateral proptosis (p< 0.02) and progression in the not operated eye followed from 6 months to 2 years period. It was reported in literature that TED has a more aggressive course in males [21], however, we were not able to demonstrate statistically the same figure.

**Table 1:** Comparison of demographic, clinical, biochemical features and different therapies between the 2 groups of patients

**Comparative values among two groups**				
	**Group 1**	**Group 2**		
	**N°(%)**	**N°(%)**	**p**	**Test**
**Sex**				
Male	25 (32.9)	6 (46.2)	***0.54***	***Χ***^***2***^
Female	51 (67.1)	7 (53.8)		
**Surgery**				
With decompression	22 (28.9)	8 (61.5)	***0.05***	***Χ***^***2***^
Without decompress.	54 (71.1)	5 (38.5)		
**Corticosteroid therapy**				
Performed	40 (52,6)	10 (76,9)	***0.14***	***Fisher***
Not done	36 (47,4)	3 (23,1)		
**Radiotherapy**				
Performed	4 (5.3)	1 (7.7)	***0.55***	***Fisher***
Not done	36 (47,4)	3 (23,1)		
**Initial thyroid hormone status**				
Euthyroid	4 (5.3)	1 (92.3)	***0.55***	***Fisher***
Hyperthyroidism	72 (94.7)	12 (7.7)		
Median age	44 (19–71)	32 (24–58)	*0.1*	*U*
CAS (mean value+/− DS)	4.2±1.8	4±0.8	0,66	*t*
**Smoker**	17 (19.)	12 (50.0)	0.02	***Χ***^***2***^

No difference of hormonal status between the 2 groups was found at the initial presentation.

## Discussion

TED usually involves both orbits, although clinical signs tend to manifest asymmetrically. When TED presents with unilateral proptosis, clinical signs and symptoms such as inflammation, impairment of eye motility and eyelid retraction [22], may be present in the fellow eye, and contralateral proptosis may become clear during the course of the disease [9,12]. Literature on actual unilateral TED is relatively scarce and heterogeneous and there are no conclusive data and explanations for this event. The number of patients having pure unilateral TED reported to range from 9% to 15% [9,10,23]. In our study, we found 13% of patients presenting unilateral TED with the appearance of unilateral proptosis. Bartley reported unilateral proptosis at the onset in 8,5% of patients in a cohort of cases with TED [24]. Kendler and Rootman reported unilateral exophthalmos in 13% of patients from a series of 557 consecutive cases [21]. This variation could be explained by the fact that in previous studies, the evaluation of proptosis was based solely on Hertel exophthalmometry, hence the different percentages reported might be explained by an unavoidable inter-observer variation when using this method [25,26]. Our study includes a review of CT imaging to more accurately define the proptosis as being asymmetric, because some reports suggest that clinically unilateral disease is bilateral from the onset [9,12]. It is also known that a CT scan can demonstrate contralateral eye muscle involvement in 50–90% of patients with clinical unilateral eye involvement [18,23]. Given the systemic nature of the disease, it seems far more likely that both orbits were involved but to a very different extent. This is supported by the fact that CT imaging picked up some sign of contralateral involvement in this study patients despite no clinical signs. Eventually, it is possible that Hertel and CT imaging measurements, though within the normal range, may be increased for an individual patient. More accurate testing, such as MR imaging, would likely show a higher number of uninvolved orbits having subtle changes, but we were unable to collect data on MRI exam on all patients.

Published data concerning delayed development of proptosis in the contralateral eye are very few. Kalmann and Mourits have described one case of late recurrence of unilateral TED on the contralateral side after 7 years of follow-up [27].

We observed 13 patients who developed a subsequent increase in exophthalmometry measurements in the non-proptotic eye within a period ranging from 6 months to 7 years. We believe that such a variable range of time for this occurrence suggests the presence of different pathological mechanisms. For those presenting with proptosis within 36 months, unilateral TED probably represents an early stage of the disease-initially limited to only one eye, and the contralateral proptosis is due to a prolonged manifestation of the disease, concealing its activity due to the use of corticosteroids and radiotherapy [9]. A high percentage of patients in both groups in our study had been treated with a corticosteroid before presentation, and it remains a challenge to explain factors leading to a less rapid progression of TED in one eye rather than the fellow eye. Considering that the 78% patients in group 2 needed corticosteroid versus the 53% of patients of group 1, it could be argued that patients who progressed onto bilateral proptosis have worse disease in general, and this could be suggested by a trend even in the absence of statistically significant p value.

When proptosis occurred within a period between 3 and 5 years, which describes 40% of patients in our study, it may be considered to be a result of subclinical, slow progressing, chronic inflammation and (or) fibrosis. We did not observe an actual increase of the CAS (4.41±1.3) before the proptosis occurred.

We observed 3 patients who had contralateral proptosis after >5 years, perhaps as a consequence of a late reactivation of TED (CAS: 5±0.786). Late reactivation of TED was also reported in 5% of patients having, with an increase in "Hertel" exophthalmometry readings after 5 years of follow-up [11]. The authors suspected that this was not due to a slow subclinical progression of disease activity or to fibrosis prior to representation, but rather due to a significant increase in activity at the time of recurrence [11]. We observed an increase of CAS during the 6 months prior to the presentation of proptosis; this observation is consistent with this proposed mechanism.

The clinical course of TED is independent of thyroid status and a temporal relation to thyroid disease is not consistent [28], but TED tends to be more severe in patients with poorly controlled hyperthyroidism and those rendered hypothyroid [11]. TED may occur under euthyroid conditions with no obvious precipitants and it often presents as a reactivation of myopathy [11]. In our cases, there was no significant difference in the initial thyroid status when comparing the 2 groups of patients. We considered only the thyroid status during the previous 6 months prior to development of the contralateral proptosis, and hence we are unable to offer statistics concerning the influence of different treatments used for thyroid disorders and TED, which may in some way affect the evolution of proptosis.

It is thought that individuals >50 years of age tend to have worse ophthalmopathy than younger individuals [23,28]. In our investigation, the risk for developing contralateral proptosis was higher in younger patients (average age of group 1 was 8.5 years less than age of group 2).This finding correlates well with the results reported by Rootman, who found that TED in young patients tends to be more asymmetric [21].

It is possible that the difference in average age between the groups could be accounted for by younger patients who tend to have a prolonged active phase (which would be more likely to progress on either side), while older patients came to our clinic in the post-inflammatory phase.

It is well known that smoking also contributes significantly to the severity and recurrence of TED [29,30]. In our study smoking history was found to be related to the development of contralateral proptosis, thus, it is important for patients with Graves’ disease to refrain from smoking.

Comparing the different therapies previously administrated (corticosteroids, radiotherapy, and surgical decompression), only surgical decompression was associated with a statistically significant occurrence of contralateral exopthalmos. We believe that this result could be related to the fact that TED is an autoimmune disease, and the releasing and spreading of an immunogenic orbital inducer during surgical decompression can serve as a trigger agent in developing contralateral proptosis. Wai et al. described a severe reactivation of TED, which occurred 3 weeks after cataract surgery in a patient who had inactive ophthalmopathy for 24 years. The authors hypothesized that trauma and pressure in the retrobulbar space induced by retrobulbar anesthsia triggered local inflammatory and immune responses, which in turn caused progression of TED [31,32]. Recently a similiar mechanism was reported to explain 3 cases of homolateral reactivation of the disease after orbital decompression [33]. Wenz et al. described 3 patients who had orbital decompression for compressive optic neuropathy and then subsequently relapsed due to progressive extraocular muscle enlargement [34].

Even if no significant difference was found for corticosteroids therapy, the low levels of p-value and power of the statistic test (0.14, power <70%) could suggest a trend of a worse disease in general for those patients who developed contralateral proptosis, since they needed corticosteroid in higher percentage.

## Conclusions

Our data supports the view that the non-proptotic and clinically less affected eye could be involved in TED during the long-term follow-up. Thus, although the clinical presentation of TED may often be asymmetric, it is believed to be truly unilateral in a minority of cases, as would be expected in a systemic disease. We believe, however, that all the hypotheses proposed to explain unilateral TED in previous studies and in the present one are still biased by the lack of a general consensus concerning the measurement values used to determine disease activity, progression, and severity. The main goal of our study was to assess the unilateral TED with the appearance of unilateral proptosis and to evaluate the occurrence of subsequent contralateral proptosis. Therefore this study would provide information necessary for the management of patients affected by TED, since we believe that the knowledge concerning the progression of unilateral presentation of TED to bilateral might be useful both for the physician when planning the therapy and for the patients.

## Consent

Written informed consent was obtained from the patient for publication of this report and any accompanying images.

## Abbreviations

TED: Thyroid eye disease; CAS: Clinical activity score.

## Competing interests

No financial support was received for this submission.

The authors declare that they have no conflict of interest.

## Authors’ contributions

DS designed the study. RP, IB, CC, MN, MF, PG, PR, PN, ADA, AI, RL conducted the study. DS, RP, AE, AB, GB took care of collection, management, analysis, and interpretation of the data. DS and MN prepared, reviewed and approved the manuscript. All authors read and approved the final manuscript.

## Pre-publication history

The pre-publication history for this paper can be accessed here:

http://www.biomedcentral.com/1471-2415/13/21/prepub
